# S-phase checkpoint regulations that preserve replication and chromosome integrity upon dNTP depletion

**DOI:** 10.1007/s00018-017-2474-4

**Published:** 2017-02-20

**Authors:** Michele Giannattasio, Dana Branzei

**Affiliations:** 1 Fondazione Istituto FIRC di Oncologia Molecolare (IFOM), Via Adamello 16, 20139 Milan, Italy; 2 Dipartimento di Oncologia ed Emato-Oncologia, Università degli Studi di Milano, Milan, Italy

**Keywords:** ATR/Mec1/Rad3, Rad53/CHK1/Cds1, Stalled replication forks, Fork remodeling, Chromosome fragility, Nucleases, Helicases

## Abstract

DNA replication stress, an important source of genomic instability, arises upon different types of DNA replication perturbations, including those that stall replication fork progression. Inhibitors of the cellular pool of deoxynucleotide triphosphates (dNTPs) slow down DNA synthesis throughout the genome. Following depletion of dNTPs, the highly conserved replication checkpoint kinase pathway, also known as the S-phase checkpoint, preserves the functionality and structure of stalled DNA replication forks and prevents chromosome fragmentation. The underlying mechanisms involve pathways extrinsic to replication forks, such as those involving regulation of the ribonucleotide reductase activity, the temporal program of origin firing, and cell cycle transitions. In addition, the S-phase checkpoint modulates the function of replisome components to promote replication integrity. This review summarizes the various functions of the replication checkpoint in promoting replication fork stability and genome integrity in the face of replication stress caused by dNTP depletion.

## Sources of DNA replication fork pausing, slow-down and arrest

DNA replication forks pause or stall at hard-to-replicate genomic regions containing natural pausing elements [1, 2], at sites containing DNA lesions [3, 4], and in the presence of DNA replication inhibitors [5, 6], such as inhibitors of dNTP pools, and drugs that inhibit replicative DNA polymerases and DNA topoisomerases (see Table 1). Numerous chemical, physical or genetic perturbations can influence the structure of specific genomic regions, induce DNA lesions, or inhibit activities required to synthesize DNA. The major categories of replication fork blocking elements, DNA lesions and frequently used DNA replication inhibitors are listed in Table 1. In this review, we summarize the main cellular responses to dNTP depletion caused by treatment with hydroxyurea (HU), a reversible inhibitor of the ribonucleotide reductase (RNR) [7]. This agent is widely used in fundamental research and as chemotherapeutic agent [8]. Mechanistically, HU quenches the tyrosyl free radical at the active site of the RNR component M2, inactivating the RNR enzyme [9, 10].

**Table 1:** DNA replication stress inducing agents

Natural pausing elements			
Non-nucleosomal DNA–protein complexes	Transcribed units (pausing elements containing DNA:RNA hybrids, R-loops)	Repeated DNA sequences	Secondary DNA structures (intra-strand DNA pairings)
Replication fork barriers (rDNA)	RNA polymerase I genes (rDNA)	TNR Trinucleotide repeats	G-quadruplex DNA structures
Centromers	RNA polymerase II genes (mRNA)	Inverted repeats	Stem loops
Telomers	RNA polymerase III genes (tRNA)	Directed repeats	Hairpin loops
Inactive DNA replication origins	TERRA transcripts (Telomeric Repeats-containing RNA)	Telomeric repeats	Triplex DNA
Heterochromatin	LTR-Long terminal repeats (transposons)	Centromeric DNA repeats	Z-DNA
Mating type *loci*	Virus or Retrovirus dependent transcripts	Satellite DNA	Cruciform structures

Lesions to the DNA bases			
Oxidation of DNA bases	Deamination of DNA bases	Methylation of DNA bases	UVC-induced photo-adducts and inter-strand crosslinks
Oxidation of Guanine to 8-hydroxyguanine	Cytosine deamination to Uracil	Cytosine methylation to 5-methylcytosine	Cyclobutane pyrimidine dimers
Oxidation of Cytosine to 5-hydroxycytosine	Deamination of 5-methylcytosine to thymine	Adenine methylation to 3-methyladenine	6–4 photoproducts
		Guanine methylation to 7-methylguanine	Nitrogen mustard or Mitomycin C-induced inter-strand DNA crosslinks
Other DNA lesions			
Abasic sites	Single strand DNA nicks	Single strand DNA gaps	Double strand breaks

DNA replication inhibitors			
Inhibitors of the cellular pool of dNTPs	Inhibitors of the DNA polymerases	Inhibitors of the DNA topoisomerases	
Hydroxyurea	Aphidicolin	Camptothecin	
Mimosine	Arabynosyl Cytosine Nucleotide analogs	Etoposide	

Several differences between HU and other types of replication perturbations are important to note here. Natural pausing elements and DNA lesions—when present at relatively low density—perturb or block progression of a subset of active replication forks [11–14], whereas HU slows the advance of the entire population of active forks, which do not necessarily encounter DNA template alterations [5]. The cellular response to these different types of DNA replication perturbations, which are detected and dealt with by DNA damage response (DDR) [15] and DNA damage tolerance (DDT) mechanisms [4], will have some unique as well as common features. In this review, we summarize S-phase checkpoint-dependent control following dNTP deprivation, focusing particularly on the knowledge derived from budding and fission yeast model systems and highlighting similarities or differences with higher eukaryotes, when studies are available. The S-phase checkpoint primarily depicted here involves the budding yeast Mec1 and Rad53 kinases corresponding to ATR and CHK1 in human cells, and Rad3 and Cds1 in fission yeast (see definition of these factors in the following section). For a summary on the DNA damage checkpoint factors, and their activation upon specific types of DNA damage, we invite the readers to other recent reviews [16, 17] and to the last section of this review. We will also provide a description of the phenotypes caused by mutations in the S-phase checkpoint pathway, with emphasis on the DNA replication fork alterations and chromosome fragmentation entailed by dNTP depletion.

The practical importance of studying the cellular responses to DNA replication fork arrest lies in the fact that many DNA replication inhibitors, such as HU, are chemotherapeutic agents [8]. Therefore, the knowledge of the underlying molecular mechanisms and responses can inform therapeutic approaches. Such knowledge could explain why cancer cells containing alterations in the ATR–CHK1 signaling pathway are selectively killed by certain DNA replication inhibitors, while cells in which this signaling is functional may show high levels of resistance [18–20]. Moreover, stalled replication forks have been shown to be potent inducers of genomic rearrangements, which are frequently associated with cancer [21–24]. Therefore, understanding the regulatory mechanisms and DNA transitions induced at stalled forks can lend important clues in the etiology of genome instability induced by specific replication stress cues.

## Replication fork-extrinsic S-phase checkpoint-dependent regulations triggered by DNA replication inhibition

S-phase checkpoint-dependent controls activated upon HU-induced replication stress do not necessarily rely on DNA replication fork components. We will refer to these regulatory mechanisms as replication fork-extrinsic controls. For instance, one of the first studied functions of the replication checkpoint relates to its role in delaying cell cycle transitions in response to certain perturbations until the initial problem is fixed [25]. *MEC1* (Mitosis Entry Checkpoint 1) in *Saccharomyces cerevisiae* (hereafter, *S. cerevisiae* or budding yeast) and *rad3* (RADiation sensitive mutant 3) in *Schizosaccharomyces pombe* (hereafter *S. pombe* or fission yeast) have been isolated as genes necessary to inhibit mitosis entry and chromosome segregation in the presence of blocked DNA replication [26, 27] (Table 2). In line with studies in yeasts, it was afterwards established that one fundamental function of their human ortholog ATR (Ataxia Telangiectasia and Rad3 related) is to prevent the onset of mitosis in the presence of irregularities during DNA replication detected by the S-phase checkpoint [28, 29]. The budding yeast Rad53 (RADiation sensitive 53), fission yeast Cds1 (Checking DNA Synthesis 1) and human CHK1 (CHeckpoint Kinase 1) kinases were then shown to have similar effects on cell cycle control following replication perturbation with HU [30–33].

**Table 2:** Key protein kinases of the Mec1^Rad3/ATR^–Rad53^Cds1/CHK1^ pathway

	*S. cerevisiae*	*S. pombe*	Human
Apical kinase	Mec1	Rad3	ATR
Effector kinase	Rad53	Cds1	Chk1

The above-mentioned serine/threonine kinases function as a hierarchical kinase pathway known as the S-phase checkpoint, in which the signal is relayed from Mec1/Rad3/ATR to Rad53/Cds1/CHK1 [31, 33–37]. The major kinases of the Mec1^Rad3/ATR^–Rad53^Cds1/CHK1^ pathway in yeast and mammalian cells are summarized in Table 2. The ways in which DNA damage and replication-associated lesions [primarily RPA-coated single stranded (SS) DNA] are recognized by Mec1/Rad3/ATR and the signal is relayed towards downstream kinases have been and continue to be intensively studied. For this topic, we invite readers to recent reviews [16, 38] and to the last section of this review. In this section, we summarize replication fork-extrinsic checkpoint-mediated regulations that affect chromosome stability via controls of cell cycle transitions, dNTP pools, origin firing, and gene gating.

**Fig. 1:**
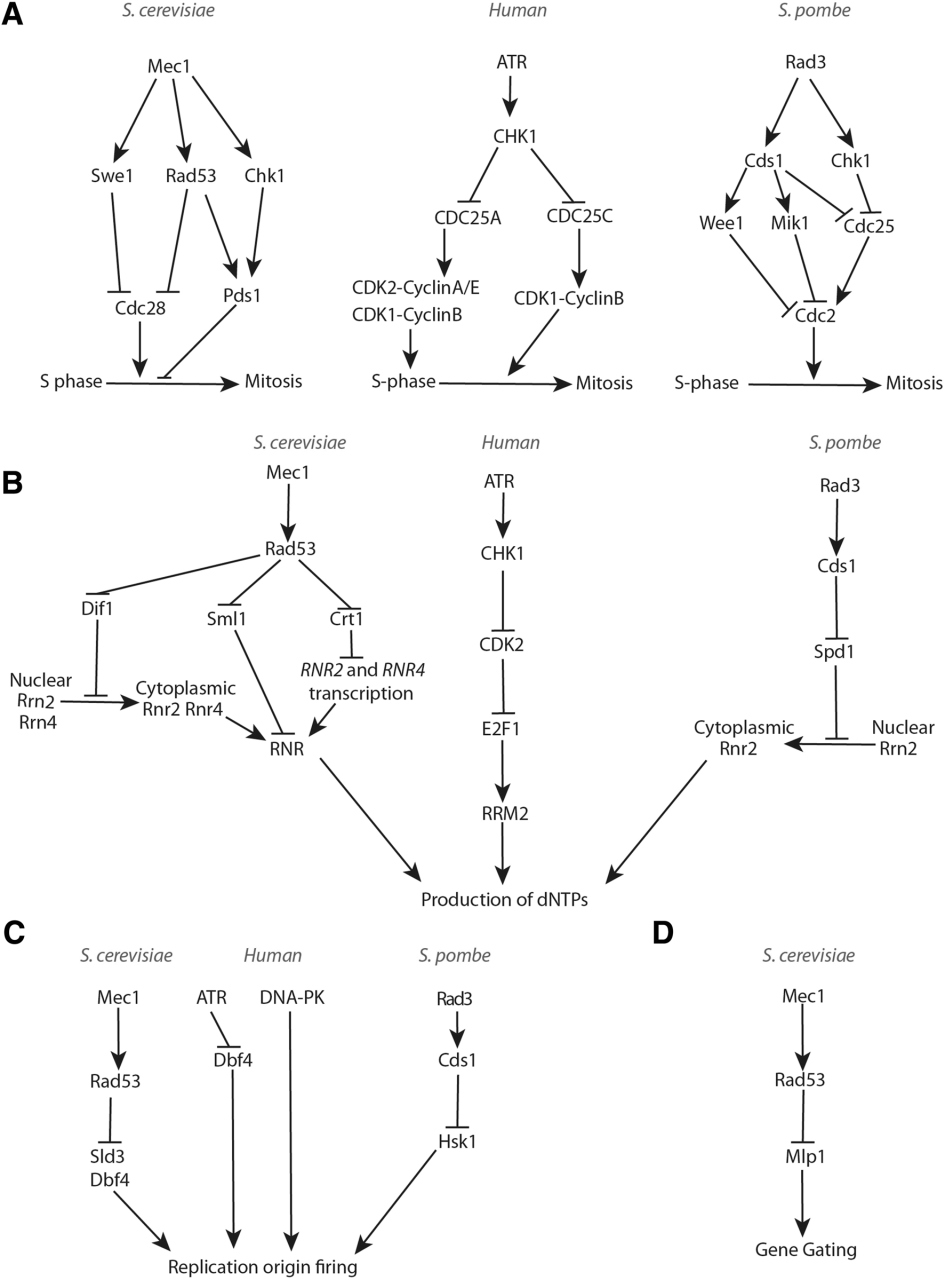
S-phase checkpoint-dependent replication fork-extrinsic controls in response to DNA replication inhibition. **a** Cellular controls that inhibit mitosis in the presence of stalled forks and incomplete DNA replication. **b** S-phase-dependent checkpoint signaling required for the up-regulation of dNTPs following DNA replication inhibition and DNA damage. **c** Molecular mechanisms underlying replication origin firing inhibition upon replication stress or blocked DNA synthesis (related to "Replication fork-extrinsic S-phase checkpoint-dependent regulations triggered by DNA replication inhibition"). **d** Checkpoint-mediated restriction of gene gating in budding yeast

Early studies suggested the notion that the S-phase checkpoint prevents entry into mitosis upon HU-induced replication perturbations. In budding yeast, upon recruitment on ssDNA–RPA complexes generated at the stalled DNA replication forks (see "Structural determinants and protein factors required for S-phase checkpoint activation in response to DNA replication stress" of this review), Mec1 activates Rad53 and the mitosis inhibitor protein kinase Swe1 (*Saccharomyces* WEe1 homologue 1), and these kinases synergistically inhibit the mitosis-promoting activity of Cdk1 (Cyclin-Dependent Kinase 1) [39]. In addition, Mec1-mediated activation of budding yeast Chk1 stabilizes the securin Pds1 (Precocious Dissociation of Sisters 1), which prevents mitotic entry by inhibiting Separase/ESP1 (Extra Spindle Pole body 1) and, subsequently, the proteolysis of cohesin, a protein complex that holds the sister chromatids together until anaphase (Fig. 1a) [40–42]. In fission yeast, Rad3–Cds1 inhibits the activity of the mitotic kinase Cdc2 (Cell Division Cycle 2) by activating mitosis-inhibitory kinases Wee1 (“wee” from small, as loss of Wee1 activity causes cells to enter mitosis before reaching the appropriate size so that cytokinesis generates abnormally small daughter cells) and Mik1 (Mitotic Inhibitor Kinase 1) that cooperate in the inhibitory phosphorylation of Cdc2 [43, 44]. In addition, Rad3 acts via Cds1 and Chk1 activation to inhibit the phosphatase Cdc25 (Cell Division Cycle 25), which can activate Cdc2 by removing the inhibitory Wee1- and Mik1-dependent phosphorylation (Fig. 1a) [45, 46]. Thus, low CDK and Cdc25 phosphatase activities, together with a high level of Securin, ensure strong inhibition of chromosome segregation in the presence of DNA replication problems detected by the S-phase checkpoint (Fig. 1a). In human cells, multiple cyclin-dependent kinases (CDKs) are present, and the basic mechanism of inhibition of mitosis entry following HU-induced replication arrest is conserved. That is, ATR/CHK1-mediated phosphorylation events cause inhibition of the CDK activators Cdc25A, Cdc25B and Cdc25C (Fig. 1a) [47].

Besides adjusting cell cycle transitions, another critical function of the S-phase checkpoint is to increase the synthesis of dNTPs. This function of the replication checkpoint was discovered in budding yeast in unperturbed conditions in a search for mutations that could bypass the lethality associated with *MEC1* deletion. Ablation of the *SML1* (Suppressor of *Mec1* Lethality 1) gene, encoding for the inhibitor of RNR (RiboNucleotide Reductase), suppresses *mec1* lethality [48]. It is now known that Sml1 is phosphorylated and degraded in a manner dependent on the kinases Mec1, Rad53 and Dun1 (DNA damage UNinducible 1) at the beginning of each unperturbed S-phase and when DNA replication is stalled (Fig. 1b) [49]. The Mec1–Rad53–Dun1 kinases also act to phosphorylate and inhibit the transcription repressor Crt1 (Constitutive RNR Transcription regulator 1) [50]. This leads to induction of the expression of several genes, including those encoding for the RNR subunits, thus providing additional means to increase the dNTP pools before the beginning of each S-phase or following DNA replication inhibition (Fig. 1b). Moreover, Mec1–Rad53–Dun1-dependent up-regulation of RNR under replication stress involves Dun1-mediated proteasome-dependent degradation of Dif1 (Damage-regulated Import Facilitator 1), responsible for nucleus-to-cytoplasm redistribution of the Rnr2 and Rnr4 subunits of RNR (Fig. 1b) [51]. A similar mechanism is at work in *S. pombe*, where Cds1 inhibits the small regulator of RNR, Spd1 (S-Phase Delayed 1), leading to the re-localization of the RNR subunits to the cytoplasm (Fig. 1b) [52]. Importantly, combined over-expression of the *RNR2* and *RNR4* genes partially suppresses the HU hyper-sensitivity of *rad53* mutant cells, supporting the idea that S-phase checkpoint-dependent RNR up-regulation contributes to cell survival of *rad53* cells under conditions that inhibit RNR [53]. Up-regulation of the cellular pool of dNTPs through the degradation of RNR inhibitors, increased transcription of the RNR genes, and subcellular re-localization of the RNR subunits, are also potent cellular responses to DNA replication inhibition in mammalian cells where the ATR–CHK1 kinase pathway induces the accumulation of the RRM2 (Ribonucleoside-diphosphate Reductase subunit M 2) subunit of RNR following replication stress (Fig. 1b) [54]. Similar to results in yeast, high levels of RRM2 were also shown to suppress different phenotypes associated with ATR dysfunction and insufficiency [55].

The S-phase checkpoint also prevents (late) origin firing when cells are faced with limiting dNTP pools. The underlying mechanism in budding yeast involves Rad53-dependent inhibitory phosphorylation of the replisome component Sld3 (Synthetically Lethal with Dpb11 3), and of the Dbf4 (DumbBell Former 4) subunit of Cdc7 (Cell Division Cycle 7)/DDK (Dbf4-Dependent Kinase), required for induction of origin firing (Fig. 1c) [56]. Recent work in mammalian cells indicated that following replication stress, inhibition of origin firing serves to indirectly protect the stalled forks by preventing exhaustion of RPA that coats ssDNA exposed at replication forks. Thus, inhibition of origin firing protects the intrinsically fragile ssDNA from being converted to deleterious double strand breaks, DSBs [57]. But is inhibition of origin firing the sole mechanism underlying the protective role of the checkpoint at stalled forks? In budding yeast, a separation-of-function allele of *MEC1* (*mec1-100*), which is defective in the inhibition of late and dormant origins firing, but is proficient in DNA replication forks stabilization, revealed that *mec1-100* cells are less sensitive to HU than *mec1* null cells suggesting that fork stabilization synergizes with origin firing regulation to preserve fork integrity and genome stability under replication stress [58].

The S-phase checkpoint was also recently shown to regulate gene gating, a process that links nascent message RNA (mRNA) to the nuclear envelope and to the nuclear pore from where it gets exported to the cytoplasm (Fig. 1d). In this process, following dNTP depletion, Rad53-dependent phosphorylation of the nucleoporin Mlp1 (Myosin Like Protein 1) blocks mRNA export and releases transcribed chromatin from the nuclear pores. This process was proposed to resolve chromosomal topological constrains that can be deleterious for the architecture of the stalled DNA replication forks [59]. Ablation of gene gating, achieved by deletion of *SAC3* (Suppressor of Actin 3), and nucleoporin Mlp1 mutants mimicking constitutive checkpoint-dependent phosphorylation alleviate *rad53* checkpoint defects [59]. Thus, Rad53-mediated DNA replication fork stabilization partly involves inhibition of gene gating.

In conclusion, there are four well-documented replication fork-extrinsic S-phase checkpoint-dependent regulations triggered by the presence of arrested DNA replication forks: (1) regulations that prevent the onset of mitosis, (2) inhibit de novo DNA replication origin firing, (3) increase the cellular pool of dNTPs, and (4) release the transcribed genes from the nuclear envelope (Fig. 1). Are these functions sufficient to explain the complex phenotypes of S-phase checkpoint mutants, or other regulatory mechanisms involving control of fork-associated DNA transitions are at play? In the next section, we review the main phenotypes of S-phase checkpoint mutants and some observations that suggest that replisome-associated factors and DNA metabolism enzymes, such as nucleases and helicases, are also under the control of the S-phase checkpoint, directly or indirectly.

## Phenotypes caused by S-phase checkpoint dysfunction in unperturbed conditions and after dNTP depletion

### Replication in the absence of the S-phase checkpoint induces chromosome fragility

Budding yeast cells allowed to replicate when Mec1 is conditionally inactivated show increased chromosome fragility, as observed by increased chromosome breakage [60]. This breakage was especially striking at late DNA replication regions defined as Replication Slow Zones (RSZs) [60, 61]. It was proposed that this function of Mec1 is conceptually related to ATR roles in counteracting fragile sites expression in mammalian cells [62]. Fragile site expression in mammalian cells is generally observed in mitosis, at certain genomic regions that replicate late and whose fragility is induced by replication inhibition with aphidicolin [63]. Chromosome fragmentation induced by the absence of Mec1 or ATR was attributed to low RNR activity: this would decrease the dNTP pool below the threshold required to sustain DNA replication fork progression, thus leading to DNA replication fork collapse and breakage at the RSZs [60, 61]. In support of this thesis, it was shown that increased RNR levels alleviate fragility both in *mec1* and ATR-depleted cells [55, 60, 61].

Upon exposure to HU, mutations in *RAD53* also cause fragility in RSZs [64], although it is not yet known whether the underlying mechanism is identical to the one observed in *mec1* mutants in unperturbed conditions [60]. Interestingly, *RAD53* ablation does not influence the basal cellular pool of dNTPs [65], but contributes to up-regulation of the RNR activity (see "Replication fork-extrinsic S-phase checkpoint-dependent regulations triggered by DNA replication inhibition"). Based on these findings, it was proposed that Rad53 up-regulates the local concentration of dNTPs at ongoing DNA replication forks [65]. This hypothesis of a local up-regulation of RNR at forks has also been recently proposed in higher eukaryotes based on the finding that CHK1 depletion in human cells does not cause a decrease in the whole cellular pool of dNTP levels [66]. Interestingly, chromosome fragmentation at RSZs in *mec1* mutants is suppressed by high HU concentrations [61], although viability is highly impaired. High HU concentrations at the beginning of S-phase cause a significant fraction of replication forks in *rad53* cells to be in an irreversible reversed or resected fork conformation close to the replication origins [64, 67], thus preventing fork breakage at RSZs (see also below). Deletion of *RRM3* (Ribosomal DNA Recombination Mutant 3), encoding a DNA helicase best known for its role in promoting replication through natural pausing sites [68, 69], also suppresses fork breakage at the RSZs in *mec1* cells [61]. This result may indicate an indirect effect of Rrm3 on dNTP levels or a completely different mechanism. *rrm3Δ* cells have elevated dNTP levels due to increased endogenous DNA damage and basal level of checkpoint activation [61, 70]. In addition, Rrm3 also functions together with other DNA metabolism factors to affect stalled replication fork architecture ([64] and see below).

How does chromosome fragility arise in the absence of Mec1, Rad53 and ATR? While various pathways are likely at work, it seems that unscheduled action of certain nucleases play an important part. The action of the fission yeast Mus81 (MMS and UV Sensitive 81) endonuclease in this process was one of the first to be documented [71, 72]. In Cds1-depleted cells, Mus81-mediated processing of stalled forks accounts in large part for the chromosome fragmentation observed [71]. Interestingly, human Mus81 was recently shown to contribute to common fragile site expression [73]. Mus81 forms an endonuclease complex with Mms4 in budding yeast (Methyl Methane Sulfonate sensitivity 4) and EME1/EME2 (Essential Mitotic structure specific Endonuclease 1–2) in mammalian cells, and processes different DNA recombination and replication intermediates [74–76]. The Mus81–Mms4 activity is enhanced in G2/M via Cdk1- and Plk1 (Polo-Like Kinase 1)-dependent phosphorylation of Mms4 [75, 77, 78]. On the other hand, the replication checkpoint Mec1–Rad53 prevents premature activation of Mus81–Eme1 during replication in yeasts and human cells [66, 71, 75, 79, 80]. In fission yeast, activation of Cds1 by HU treatment induces Cds1-dependent phosphorylation of Mus81, and subsequent dissociation of Mus81 from chromatin [72]. Thus, the S-phase checkpoint protects the integrity of stalled DNA replication forks not only by regulating fork-extrinsic cellular processes (see Sect. 2), but also by regulating the spatiotemporal dynamics of nucleases, such as Mus81–Mms4 [77].

In line with the above-mentioned mechanism of chromosome fragility, Mus81- and Mre11 (Meiotic REcombination 11)-dependent DNA breaks have been recently shown to be induced in human and hamster cells in unperturbed conditions when CHK1 is ablated, confirming that one important function of the S-phase checkpoint is to prevent enzymatic activities that can cleave stalled replication forks [66]. Currently, it is not clear whether checkpoint-mediated restriction of Mus81 actions happen at specific genomic regions or at a certain time during replication. Moreover, the location of RSZs and fragile sites induced by dysfunctions in the Mec1^Rad3/ATR^–Rad53^Cds1/CHK1^ checkpoint pathway is only partly understood, although recent efforts promise to map those genomic sites on human chromosomes using quantitative genome-wide high-resolution techniques.

### S-phase in the presence of low HU concentrations induces massive chromosome fragmentation in *rad53* cells

Cells deleted for Rad53 but kept alive by the *SML1* deletion (*rad53 sml1*) show massive chromosome fragmentation when replicating in the presence of low concentrations of HU [61, 64]. Under these conditions, chromosome breakage is observed 3–5 h from the release of cells into S-phase, when bulk replication is nearly complete in wild-type cells [64]. Notably, high HU concentrations do not induce massive chromosome fragmentation in *sml1 rad53* cells, even after long incubation in HU [61]. The exact relationship between HU concentrations, time of exposure to HU in S-phase and chromosome fragmentation in *rad53* mutant cells is not completely understood, but several observations brought insights in this process. Exposure to high HU concentrations at the beginning of the S-phase strongly impedes fork progression in *rad53* defective cells, causing a high percentage of forks to be arrested in a reversed or resected fork conformation close to the DNA replication origins [64, 67]. Such alterations in replication fork structure are largely irreversible, as judged from the inability of *rad53* cells to re-start DNA replication after HU removal [81]. Thus, it is possible that reversed forks can stabilize arrested forks against breakage (see also "S-phase checkpoint roles in fork architecture: prevention of pathological DNA transitions or resolution of transient DNA intermediates?"). Alternatively, and perhaps more likely, if fragility is preferentially induced in RSZs located in late replicating regions, inhibiting replication early on will prevent replication forks to reach late-replicating genomic regions. Importantly, chromosome breakage in *mec1* cells does not require metaphase to anaphase transition, but involves condensation and Topoisomerase II-mediated activities [82]. Whether chromosome fragmentation in *rad53* cells exposed to low HU concentrations occurs through the same mechanism as the one observed at RSZs in *mec1* cells in unperturbed conditions [60, 61, 64] remains to date unclear.

### S-phase in presence of high HU concentrations in *rad53* cells alters replication fork architecture and inactivates replication

**Fig. 2:**
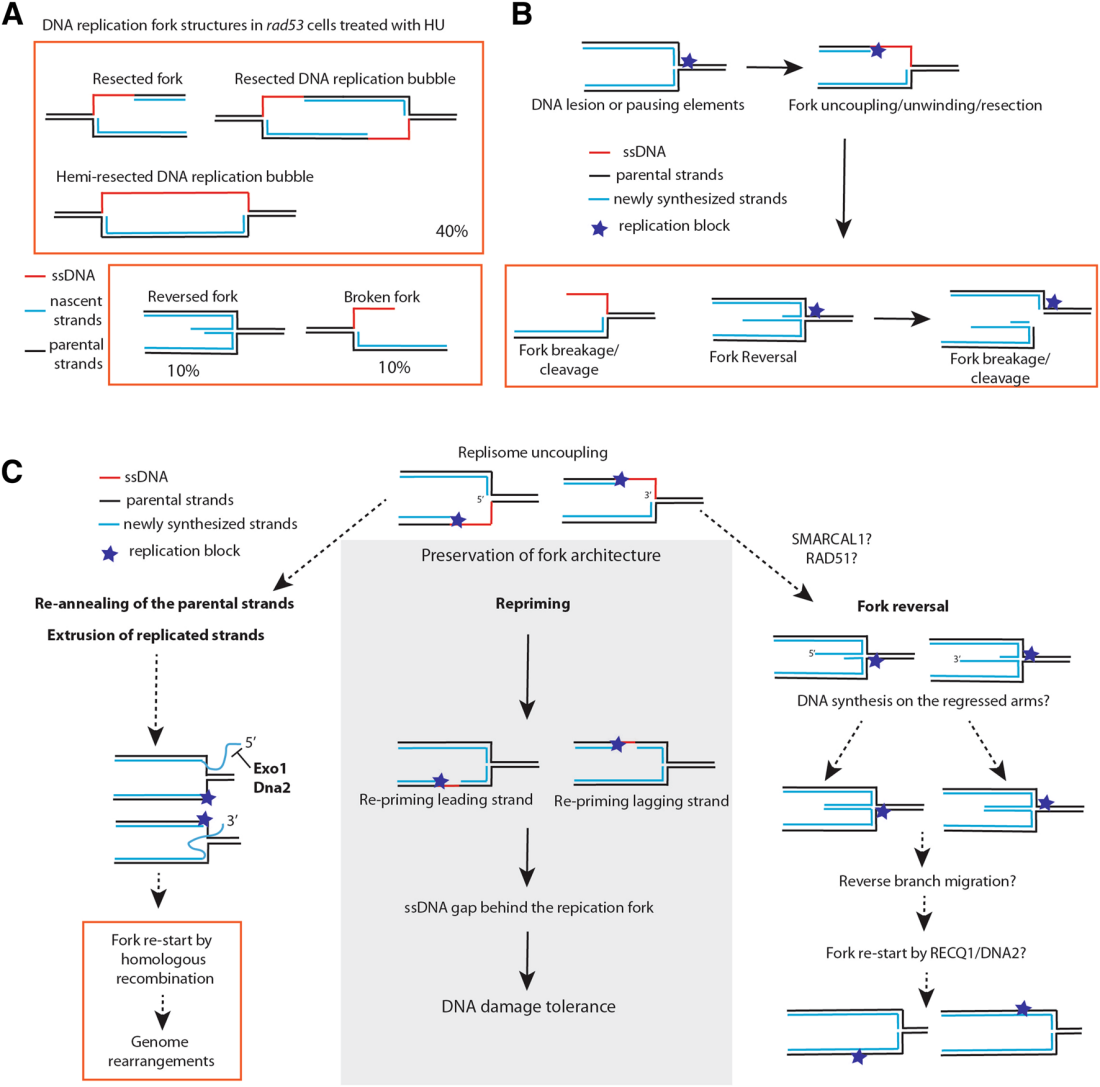
DNA replication fork alterations in checkpoint mutants and mechanisms contributing to proficient DNA replication. **a** DNA replication fork alterations (resected forks and reversed forks) accumulating in *rad53* mutants of *S. cerevisiae* treated with hydroxyurea. The relative percentage of each major DNA replication fork intermediate is shown based on previously published results [64, 67]. **b** Replication stress induces uncoupling events between leading and lagging strands. Subsequent re-annealing of the parental and nascent strands can promote structural transitions at the stalled replication forks. Processing of the intermediates can also cause chromosome breakage. **c** Cellular mechanisms for fork stabilization and re-start. Re-priming coupled to DNA damage tolerance can preserve the normal DNA replication fork architecture. DNA replication inhibition and DNA lesions can induce fork uncoupling, formation of long ssDNA stretches, long DNA flaps and fork reversal. Activities that are potentially implicated in processing of flaps and reversed forks are shown (related to "Phenotypes caused by S-phase checkpoint dysfunction in unperturbed conditions and after dNTP depletion", "S-phase checkpoint roles in fork architecture: prevention of pathological DNA transitions or resolution of transient DNA intermediates?" and "S-phase checkpoint-dependent phosphorylation events at stalled replication forks" of the review)

Structural analysis of DNA replication forks through neutral–neutral 2D gel electrophoresis and transmission electron microscopy of *rad53-K227A* kinase-defective mutant and *rad53 sml1* cells treated with high HU concentrations revealed that around 40% of forks had extensive resection (with an average of 0.8–1 Kbp of ssDNA on one of the newly synthesized strands close to the fork junction), 10% of forks had breaks, and 10% had reversed forks (Fig. 2a) [64, 67]. The ssDNA discontinuities at the fork in *rad53* cells appear to be localized on only one of the two newly synthesized strands. Moreover, a consistent fraction of resected replication forks (5%) are in a “bubble conformation” with one side of the replication bubble, with a length up to 2 Kb, being completely single stranded. These latter replication fork structures have been called hemi-resected DNA replication bubbles or hemi-replicated DNA structures (Fig. 2a) [64, 67]. Such structures are not observed in control wild-type cells, which also show a very low level of reversed forks (less then 1% of the total forks), and usually have ssDNA stretches of less than 0.2 Kb at the fork junction [67]. These results suggest a protective action of the S-phase checkpoint on the structure of stalled DNA replication forks.

The extensive resection processes observed on either leading or lagging strands in *rad53* cells [64, 67] could be explained by high frequency of resection/unwinding events of one of the two newly synthesized strands, or, alternatively, by extensive uncoupling between leading and lagging strands (Fig. 2b). It is possible that in resected and uncoupled forks, the parental strands could re-anneal causing extrusion of the newly replicated strand (either with 5′ or 3′ end) (Fig. 2b, c). Further annealing of the extruded nascent strands could induce the formation of a reversed fork with a protruding ssDNA end on the regressed arm (Fig. 2b, c). Stalled forks of *rad53* cells may undergo complete elimination of leading and lagging strand filaments, causing formation of hemi-resected DNA replication bubbles (Fig. 2a). We note that DNA replication forks with extended regions of ssDNA or reversed forks carrying single Holliday Junction (sHJ) centers may undergo spontaneous or nuclease-mediated processing with the formation of DSBs, thus representing a potential source of chromosomal rearrangements and genome instability [80, 83, 84] (Fig. 2b).

### Controversial roles of the S-phase checkpoint on replisome maintenance and association with stalled replication forks

S-phase checkpoint mutants exposed to high concentrations of HU were shown to undergo progressive dissociation of the replicative DNA polymerases from early ARS regions containing replication-derived forks [64, 85, 86]. However, this notion has become somewhat controversial. Chromatin immunoprecipitation studies reported decreased binding of Polα at early active Autonomously Replicating Sequences (ARSs) of *rad53* cells treated with high HU concentrations [64, 85–87]. However, another report concluded that Polα dissociation in *rad53* cells only takes place at a small subset of forks localized at very early ARS regions [88]. In this latter study, the authors purified the replisomes from HU-treated *rad53* and wild-type cells, revealing the presence of fully assembled replisomes in the absence of Rad53. The replisome composition was not changed, but whether the purified replisomes were still active and associated to the forks in vivo is not yet known, in spite of the multiple efforts dedicated by the authors to elucidate confounding effects [88]. Moreover, purification of the total pool of replisomes at a given time can be influenced by the presence of functional replisomes coming from de novo origin firing, a process that is deregulated in *rad53* mutants. Furthermore, control cells may undergo DNA replication termination faster, which would cause dissociation of the replisomes from the chromosomes. These factors may potentially mask the differences between wild-type and *rad53* cells, in which replication is slower than in wild type in the presence of HU. Similar experiments on the replisome composition were conducted in human cells, and the results confirmed that the replisome associates normally to the nascent strands in ATR-inhibited cells exposed to HU [89].

Various studies indicate that cells with non-functional checkpoints have a different replication fork architecture in comparison with wild-type cells (see Sect. "S-phase in presence of high HU concentrations in *rad53* cells alters replication fork architecture and inactivates replication") and accumulate Rad52 (RADiation sensitive 52) recombination protein foci in S phase [90, 91]. Importantly, *mec1* cells under replication stress strongly depend for viability on factors with roles in homologous recombination, such as Rad52 [92] and the RecQ helicase Sgs1 (Slow Growth Suppressor 1) [93]. However, Rad52 also has annealing activity, and therefore its requirement for viability in *mec1* cells may reflect increased annealing events triggered by elevated levels of ssDNA during replication, similar to a situation recently reported in Polymerase α/Primase mutants [94].

How these apparently contradictory results on replisome composition and DNA polymerase association in checkpoint defective cells can be explained remains still puzzling. Hopefully, future research using conditional inactivation of S-phase checkpoints and advanced genomic and visualization techniques will illuminate the kinetics to which different replisome and recombination factors associate to replication forks in checkpoint proficient and deficient cells, and will shed new light on the effect of specific replisome-mediated processes to the complex phenotypes of checkpoint mutants.

## S-phase checkpoint roles in fork architecture: prevention of pathological DNA transitions or resolution of transient DNA intermediates?

A prominent phenotype of S-phase checkpoint mutants exposed to dNTP depletion is an altered replication fork architecture—compared to the one of wild-type cells, characterized by increased fork reversal and resection of newly synthesized strands [60, 67, 81] (see also "S-phase in presence of high HU concentrations in *rad53* cells alters replication fork architecture and inactivates replication"). The structures accumulating in *rad53* cells treated with HU could represent either pathological intermediates that are actively prevented by the replication checkpoint, or normal transient structures that are not detectable in wild-type control cells because their processing or resolution might rely on the replication checkpoint [95]. Whether fork reversal is actively prevented or not by the S-phase checkpoint is an important notion to discuss, as this has general implications on the roles of reversed fork intermediates for replication and genome stability. These roles have remained controversial and a matter of debate.

Recently, it was proposed that reversed forks are central intermediates of replication fork stabilization and re-start mechanisms under replication stress, based on the observation that mammalian cell lines exposed to different sub-lethal doses of replication stress-inducing agents activate a RAD51 (the ortholog of budding yeast RADiation sensitive 51)-dependent pathway that promotes formation of reversed forks [96]. In this view, when fork progression is challenged, RAD51-dependent reactions would convert stalled forks into reversed forks [96]. RECQL1 (RECQ Like helicase 1) helicase and DNA2 (DNA Replication Helicase/Nuclease 2) nuclease were proposed to subsequently process and restart the reversed forks (see Fig. 2c) [97–99]. Several questions remain, however, open about the mode of action of the RAD51–RECQ1–DNA2 pathway of fork stabilization and re-start through fork reversal. For example, replisome location and the relationship between the replisome and the replication fork during the formation of the reversed fork and its re-start are not well defined. Although human RAD51 plays a role in protecting the nascent stands of replication forks from MRE11-dependent resection in unperturbed conditions [100], the exact roles of RAD51 in DNA replication in general and in reversed fork formation following replication stress in particular are still under investigation. Does fork reversal lead to a replisome-dependent fork restart? Is RAD51-mediated fork reversal the best option for fork reactivation or is it a last-resort option? Is fork reversal triggered genome-wide or is preferentially induced at specific genomic regions where other fork reactivation mechanisms fail?

The current insufficient knowledge of factors processing reversed forks and the lack of techniques that can map single-ended DSBs on the chromosomes do not allow precise answers to the above questions. However, it is useful to consider what other mechanisms may mediate fork restart independently of fork reversal. One such mechanism involves replicative helicase-coupled re-priming downstream of the stalled replisome, to allow re-initiation of DNA synthesis after the replication obstacle (Fig. 2c) [94, 101]. This would preserve a normal replication fork structure and induce formation of DNA gaps, which could be filled-in postreplicatively [102, 103]. While this mechanism has been primarily studied in the context of DNA damage tolerance induced by alkylating agents, in principle it can operate in response to other types of replication obstacles or replication stress cues that do not block Polα–Primase activity. Notably, additional specific DNA polymerases directing re-priming events at stalled forks are starting to be identified in mammalian cells [104, 105], suggesting that even in conditions when Polα–Primase activity is inhibited, re-priming events may be induced.

Interestingly, most processes related to replication intermediate metabolism and the function of the replication checkpoint are conserved from yeast to mammals, but fork reversal is much more frequent in mammalian cell lines than in wild-type yeast cells [67, 95, 96, 102]. We recently proposed that this observation holds insights about the contexts in which fork reversal is triggered [4]. The high complexity of the human genome, which is enriched in repetitive sequences and heterochromatic regions, may account for numerous physical or topological fork barriers that would be more easily accommodated by fork reversal rather than other fork reactivation events, such as the re-priming mechanism discussed above. Moreover, the genomic context in which these fork-stalling events happen would not necessarily trigger checkpoint activation [106]. Indeed, stalled forks at ribosomal DNA in budding yeast, the locus most abundant in repetitive sequences in this organism, do not mount checkpoint activation [107], but trigger the formation of sHJs that most likely represent reversed forks [94, 108]. We propose that the reversed forks detected in mammalian cells may often originate from repetitive sequences that represent natural obstacles for replication forks, and which may be further destabilized by treatment with replication inhibitors, such as HU [14]. In these contexts, fork reversal may promote fork stabilization until an incoming fork reaches the region. In this view, the stalled fork will not necessarily be an intermediate in the restart process, but it will represent an important strategy, present from yeast to mammals, to promote fork stability in specific genomic contexts that constitute natural replication obstacles [4].

In S-phase checkpoint mutants treated with HU fork reversal is increased [64, 67], but how does the S-phase checkpoint regulate fork reversal? Is it because other fork restart mechanisms, such as re-priming, are impaired in the absence of the S-phase checkpoint, or because the S-phase checkpoint counteracts fork remodeling or promotes resolution of the reversed fork? Is fork remodeling related to the extensive resection events observed in checkpoint mutants? Some answers began to emerge. First, supporting the view that the fork remodeling and resection events are related to each other, deletions of genes encoding the DNA helicases Pif1 (Petit Integration Frequency 1) and Rrm3 were shown to reduce the formation of both resected and reversed forks in *rad53* cells treated with HU [64]. Regarding the etiology of fork reversal, the human DNA translocase SMARCAL1 (SWI/SNF-related Matrix-associated Actin-dependent Regulator of Chromatin subfamily A-Like protein 1) was shown to induce fork remodeling [109]. Interestingly, the Pif1 DNA helicases and SMARCAL1 associate to stalled replication forks and nascent strands also in Rad53 and ATR proficient cells, respectively, but they do not exert their activities on changing the fork structure [64, 109]. Importantly, ablation of SMARCAL1 or Rrm3/Pif1 DNA helicases suppresses chromosome fragmentation in ATR and Rad53 deficient cells, respectively, suggesting that fork reversal may be a toxic replication intermediate in checkpoint mutants and subsequently induce chromosome fragmentation.

As discussed in "Replication in the absence of the S-phase checkpoint induces chromosome fragility", a significant part of chromosome breakage observed in S-phase checkpoint cells can be attributed to the unscheduled action of the Mus81 endonuclease. In addition to Mus81-Mms4/Eme1, endonuclease activity-containing factors, such as SLX4 [Synthetic Lethal of unknown (Xfunction 4] and CtIP (Carboxy-terminal Interacting Protein) are partly responsible for chromosome fragmentation in cells depleted for ATR and exposed to replication stress [109, 110]. Exo1 nuclease resects stalled and reversed forks in *rad53* cells treated with HU, and the nuclease activity of Dna2 counteracts fork reversal in fission yeast through the processing of fork-associated DNA flaps (Fig. 2c) [111–113]. Explicitly, Dna2 nuclease may reduce the length of the DNA flaps caused by extended replication fork uncoupling events (see Fig. 2c) [111]. This action will contribute to limit subsequent re-annealing of the parental strands and the extrusion of the newly synthesized filaments as 5′ or 3′ DNA flaps (Fig. 2c), thus counteracting fork reversal [111]. In this vein, an Exo1–Dna2–Sae2-dependent nuclease pathway was recently shown to counteract formation of unusual DNA replication intermediates in checkpoint defective cells exposed to replication stress [114].

In checkpoint mutants such as *rad53*, fork reversal is accompanied by increased uncoupling between leading and lagging strands, which could subsequently lead to fork reversal. Does the checkpoint prevent this uncoupling? Intriguingly, it was recently shown that HU treatment leads to the unloading of PCNA specifically from the lagging strand of the DNA replication fork [115]. As uncoupling between leading and lagging strands is not extensive in wild-type cells [67], these findings suggest that, in some way, lagging strand activities must be inhibited following DNA replication fork stalling induced by dNTP deprivation. Notably, the unloading of PCNA is mediated by Elg1 (Enhanced Level of Genomic instability 1) [116–118], which is phosphorylated by the checkpoint [119]. Thus, the extensive uncoupling of leading and lagging strands in *rad53* cells may also illustrate that Rad53 inhibits lagging strand elongation following HU-induced fork stalling. The substrates involved may relate to Elg1-mediated PCNA unloading [115], involve counteraction of Rrm3 and Pif1 [64], downregulation of DNA primase [120], and/or additional mechanisms.

**Fig. 3:**
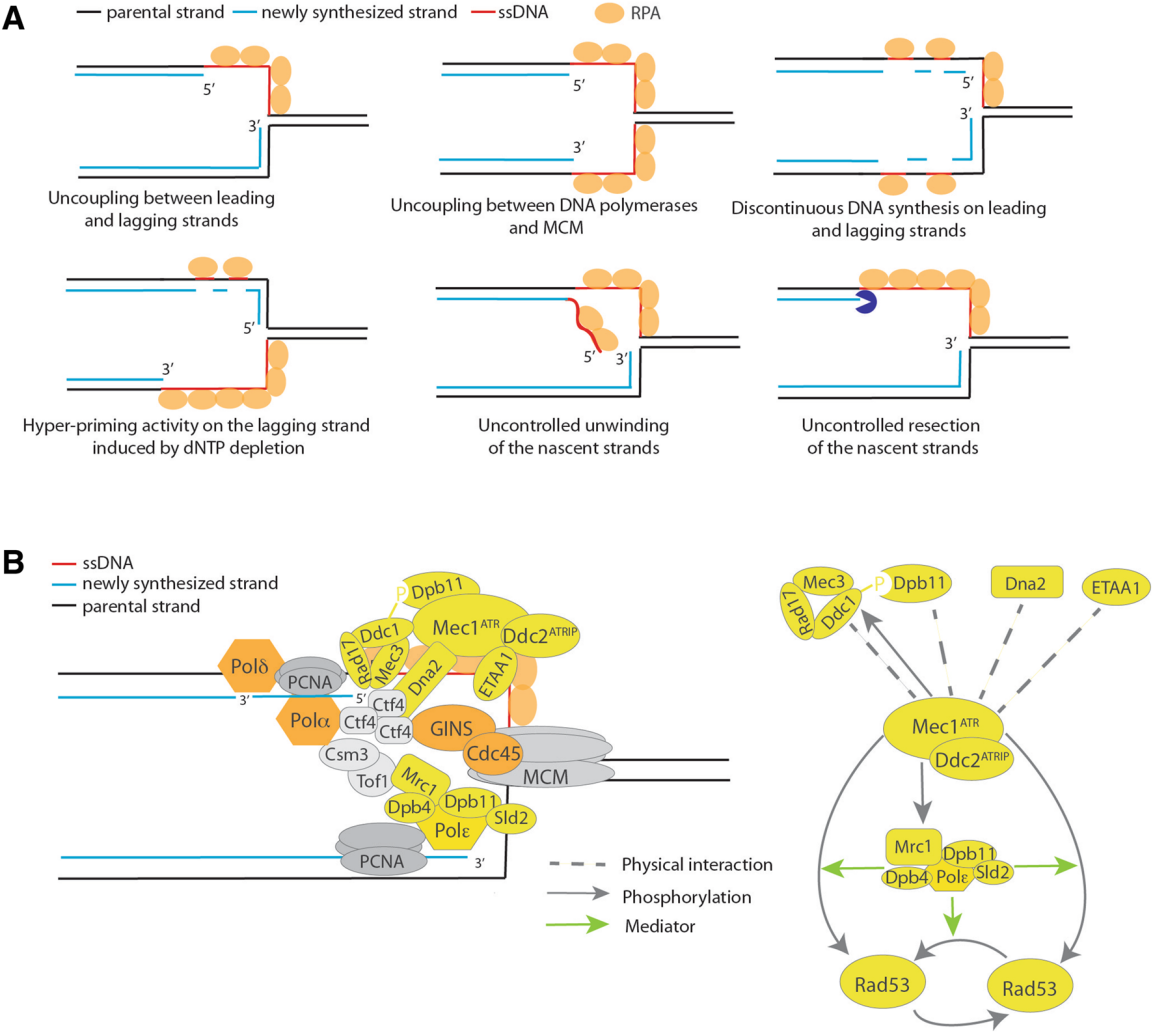
DNA substrates and protein factors required for S-phase checkpoint activation. **a** High amounts of ssDNA–RPA complexes at stalled forks and primer–template substrates can be induced by uncoupling of leading and lagging strand DNA synthesis, uncoupling between DNA polymerases and MCM DNA helicase, by discontinuous synthesis of the nascent strands, by hyper-priming activity of Polα, unwinding or resection of one of the nascent strands. **b** Simplified representation of the replication fork and some replisome components. Protein factors shown in *yellow* are involved in the activation of the Mec1^Rad3/ATR^–Rad53 ^Cds1/CHK1^ checkpoint pathway following dNTP deprivation. Physical and functional interactions instrumental to checkpoint activation are indicated through *arrows* and *dashed lines*, respectively (related to "Structural determinants and protein factors required for S-phase checkpoint activation in response to DNA replication stress")

Uncoupling between the replicative DNA helicase MCM (MiniChromosome Maintenance) complex and DNA polymerases strongly activates ATR in Xenopus egg extracts [121]. Such uncoupling is expected to generate ssDNA regions at the fork junction on both replicating strands. It was proposed that one important function of the replication fork pausing complex Tof1–Csm3–Mrc1 (Topoisomerase I interacting Factor 1-Chromosome Segregation in Meiosis 3-Mediator of the Replication Checkpoint 1) in *S. cerevisiae*, composed of Swi1–Swi3 (Switchable 1–3) and Mrc1 in *S. pombe*, and TIMELESS–TIPIN and Claspin in mammalian cells, is to maintain the coupling between the DNA synthesis apparatus of the replisome and the MCM DNA helicase (see Fig. 3b) [122, 123]. We note that predicted fork structures with ssDNA on both replicated arms have not been observed in *rad53* checkpoint mutants [64, 67] or in Xenopus egg extracts depleted for Tipin [124], potentially due to redundancy in factors ensuring the coordination between the replicative helicase and the replisome. Such factors, bridging the replisome and the replicative helicase, include Ctf4 (Chromosome Transmission Fidelity 4)/AND-1 (Acid Nucleoplasmic DNA binding protein 1) and MCM10 (MiniChromosome Maintenance 10) [125–127]. Intriguingly, in Tipin-depleted Xenopus extracts and *ctf4* single mutants in budding yeast, there is an increase in fork reversal, suggesting that failure to coordinate replisome and helicase movements may also induce alternate fork response pathways that involve formation of reversed forks [94, 124].

Taken together, these observations illustrate that unscheduled fork remodeling, resection, cleavage, and unwinding can induce cytotoxicity and genome instability in cells defective in the replication checkpoint. Recent studies suggest that many of these activities are counteracted by the replication checkpoint via phosphorylation events [64, 72, 109]. Relevant phosphorylation substrates of the checkpoint at stalled replication forks are discussed in the next section.

## S-phase checkpoint-dependent phosphorylation events at stalled replication forks

Based on the phenotypes of S-phase checkpoint mutant cells and the DNA structures arising in mutants of the S-phase checkpoint ("S-phase checkpoint roles in fork architecture: prevention of pathological DNA transitions or resolution of transient DNA intermediates?"), we pinpointed possible roles of the checkpoint in controlling the activity of replication fork components or regulators, such as nucleases and helicases. Here, we plan to discuss various studies relevant to this concept and to highlight critical substrates that emerged.

HU induces Mec1-dependent hyper-phosphorylation of the subunit 2 (RPA2) of the ssDNA-binding protein RPA (Replication Protein A) in *S. cerevisiae* [128]. Besides its roles in stabilizing the replisome and the ssDNA generated during DNA replication, the RPA complex functions as a platform to recruit ATR–ATRIP (ATR Interacting Protein) checkpoint complexes at lesion sites and at stalled forks (see also "Structural determinants and protein factors required for S-phase checkpoint activation in response to DNA replication stress"). ATR-dependent phosphorylation of RPA2 following dNTP deprivation has been shown to occur also in human cells, where this modification is critical to sustain DNA synthesis and DNA replication fork re-start, and to recruit PALB2 (PArtner and Localizer of BRCA2) to stalled DNA replication forks [129, 130]. Early studies in *S. cerevisiae* suggested that Rad53-mediated targeting of the Pri1 subunit of DNA primase (encoded by the *PRI1* and *PRI2* genes) facilitates slow-down of replication in the face of replication stress [120]. Although the molecular mechanism and the phosphorylation sites implicated in this regulation are not known, it is conceivable that such regulation of the primase activity may serve to prevent uncoupling between leading and lagging strands synthesis in the presence of replication stress (see Fig. 3a).

Chromosome breakage arises in cells depleted for or mutated in the replication checkpoint ("Phenotypes caused by S-phase checkpoint dysfunction in unperturbed conditions and after dNTP depletion"). In fission yeast and human cells, the chromosome fragmentation of cells lacking Cds1 or depleted for CHK1 largely depends on Mus81 ([66, 72], see also "Phenotypes caused by S-phase checkpoint dysfunction in unperturbed conditions and after dNTP depletion"). As mentioned in "S-phase checkpoint roles in fork architecture: prevention of pathological DNA transitions or resolution of transient DNA intermediates?", processing of the nascent strands in checkpoint mutants under replication stress can be deleterious. Exo1 is a 5′–3′ exonuclease/5′ flap-endonuclease and plays a role in the resection of the stalled and reversed forks forming in *rad53* cells exposed to HU [112, 113]. *EXO1* deletion in budding yeast does not suppress the HU hypersensitivity of *rad53* cells treated with HU, suggesting that Exo1-dependent resection of the stalled forks is an event that occurs when the structure of the fork has been already altered in an irreversible way [112]. Exo1 is hyper-phosphorylated upon HU treatment in a Mec1-dependent manner [131], but whether this serves to inhibit Exo1 activity or to regulate its cellular localization is not yet clear. In human cells, ATR-dependent phosphorylation of EXO1 leads to its polyubiquitylation and subsequent proteasome-mediated degradation, highlighting another important mechanism through which the S-phase checkpoint limits fork-processing activities [132, 133].

A number of factors have been implicated in fork remodeling. One such factor, SMARCAL1, which can promote fork regression in vitro, is phosphorylated by ATR upon replication stress [109, 134]. ATR-dependent regulation of SMARCAL1 is thought to inhibit SMARCAL1-dependent fork remodeling-induced by dNTP deprivation, and to prevent subsequent SLX4- and CtIP-dependent processing of the fork structures [109]. This finding provides support to the idea that proteins that potentially remodel, cut or resect the stalled fork are efficiently inhibited by the S-phase checkpoint. In this vein, Rad53-dependent phosphorylation of the DNA helicases Rrm3 and Pif1 at the stalled forks prevents the accumulation of both resected and reversed forks, as well as the chromosome fragmentation phenotype typical of *rad53* cells [64]. Whether Rrm3 specifically localizes to leading or lagging strands replisomes is not known, while Pif1 was proposed to participate in an alternative pathway of Okazaki fragment processing to stimulate DNA polymerase δ-dependent strand displacement activities on the lagging strand [135]. Intriguingly, human Pif1 can unwind synthetic DNA structures resembling stalled DNA replication forks and catalyze in vitro reactions that are similar to the ones thought to be involved in the formation of reversed forks [136]. Upon HU treatment, Rad53 phosphorylates Pif1 and Rrm3 [64]. Genetic data indicate that Rad53-mediated phosphorylation of Pif1 and Rrm3 counteract fork remodeling leading to fork reversal [64]. However, due to the pleiotropic effects of yeast and human cells defective in replication checkpoint function, it is difficult to derive interpretations of protein function in a checkpoint-proficient context based on phenotypes observed in checkpoint deficient cells. Substantiating the notion that helicases often act at stalled forks, in addition to the SMARCAL1, Pif1 and Rrm3 helicases mentioned above, the human FBH1 DNA helicase has also been recently shown to catalyze regression of the stalled forks following replication stress [137].

In the process of annealing of the parental strands at uncoupled forks, long 5′ flaps may be generated. Such flaps would require processing by Rad27, Exo1 and Dna2 (Fig. 2c). If long 5′ flaps on Okazaki fragments fail to be cleaved, they can induce formation of reversed forks. The notion that Dna2 nuclease deals with a toxic substrate generated by Pif1 was suggested by the observation that the lethality caused by the absence of Dna2 is suppressed by ablation of Pif1 [135]. Since Pif1 is thought to create DNA flaps at the lagging strand of replication forks during the alternative pathway of Okazaki fragment processing [138], the observed genetic interaction supports the idea that long 5′flaps at forks are toxic and counteracted (Fig. 2c).

The replication checkpoint also targets replisome components. The MCM2 subunit of MCM (Minichromosome Maintenance Complex) is phosphorylated in an ATR-dependent manner following replication stress and this event facilitates robust activation of the intra-S checkpoint [139, 140]. Thus, transmission of the S-phase checkpoint signal downstream of ATR may involve ATR-dependent phosphorylation of a series of replisome components. Psf1 (Partner of Sld Five) subunit of the GINS (Go-Ichi-Ni-San) complex of the replisome is also phosphorylated in a Mec1-dependent manner, but the physiological role of Psf1 modification is not yet known [88].

ATR also mediates the transient association of FANCD2 (FANConi Anemia Complementation Group D2) to the MCM helicase complex at stalled replication forks, although it is not known whether this is related or not to the MCM2 phosphorylation event described above [141]. FANCD2 plays roles in protecting the stalled replication fork and in restraining DNA replication after removal of HU [141]. Intriguingly, FAN1 (Fanconi Anemia associated Nuclease 1), a 5′ flap endonuclease implicated in ICL (Inter-strand CrossLink) repair and identified as interacting factor of FANCD2 [142, 143], is also recruited to stalled replication forks through its interaction with the monoubiquitylated form of FANCD2. FAN1 recruitment with FANCD2 at stalled DNA replication forks is necessary to re-start DNA replication and to prevent chromosome abnormalities even in the absence of ICLs [141, 144, 145]. Whether these actions of FAN1 following dNTP deprivation or its recruitment to FANCD2 are regulated by ATR remains still unknown.

BLM helicase, the human orthologue of *SGS1* mutated in the cancer-prone Bloom syndrome, interacts with stalled replication forks and is phosphorylated in an ATR-dependent manner following dNTP depletion, suggesting possible functional crosstalk between ATR and BLM at stalled replication forks [146]. Moreover, ATR-dependent phosphorylation of BLM is required for DNA replication fork restart and suppression of new origin firing [147].

Although already complex, it is likely that the picture of S-phase checkpoint replisome substrates will expand in the future, giving a better view of the DNA transitions that occur at stalled forks and the processes underlying fork stabilization, collapse and restart.

## Structural determinants and protein factors required for S-phase checkpoint activation in response to DNA replication stress

### DNA structures and protein signals required for S-phase checkpoint activation

The DNA damage and replication checkpoint is activated by abnormalities in the DNA, both in terms of the substrate per se and the amount of substrate generated. Early studies in yeast revealed that processing of uncapped telomeres caused checkpoint activation and that the extent of Rad53 activation during the repair of a single site-specific and non-repairable DSB correlated with the extension of resection [148, 149]. These findings suggested that non-physiological high levels of ssDNA represent a signal for DNA damage checkpoint activation. This concept was later substantiated by findings that checkpoint activation after UV irradiation in non-replicating yeast cells depends on lesion processing and exposure of ssDNA gaps [150]. Thus, uncoupling of leading and lagging strands (or of DNA polymerases and MCM helicase), due to prolonged stalling or re-priming events downstream of the lesion, can provide substrates for checkpoint activation at replication forks (Fig. 3a) [13, 121, 151]. These events could also be induced by dNTP deprivation or other treatments that inhibit DNA replication without causing DNA lesions. Further studies revealed that checkpoint activation requires recruitment of a subset of checkpoint factors, called “sensors” to the damaged sites, and this is mediated by ssDNA coated with RPA [152, 153]. The sensors include ATR–ATRIP and corresponding orthologs (Mec1–Ddc2 in budding yeast and Rad3–Rad26 in fission yeast) and the PCNA-like checkpoint clamp complex 9–1–1 [154]. 9–1–1 stands for Rad9–Rad1–Hus1 (RADiation sensitive 9–RADiation sensitive 1–HydroxyUrea Sensitive 1) in *S. pombe* and human cells, and its equivalent in *S. cerevisiae* is Rad17–Mec3–Ddc1 (Radiation sensitive 17–Mitosis Entry Checkpoint 3–DNA Damage Checkpoint 1) [155–158]. However, recruitment of 9–1–1 requires not only the presence of RPA-coated ssDNA, but also a primer–template junction, where the 5′-end of an annealed DNA fragment (primer) is close to a stretch of ssDNA [159]. Thus, continued primer synthesis at stalled replication forks can contribute to checkpoint activation [151]. Accordingly, early studies showed that decreased levels of dNTPs induce continuous synthesis of primers in an in vitro system with immuno-purified yeast DNA polymerase I and DNA primase [160]. Thus, discontinuous DNA synthesis on the nascent strands, uncoupling between leading and lagging strand DNA synthesis, and unwinding/resections events of the newly synthesized filaments induce the formation of substrates required for the recruitment of checkpoint sensors at stalled replication forks (Fig. 3a).

### Mediators of S-phase checkpoint activation following dNTP depletion

The recruitment of sensors to damaged or stalled DNA replication forks is not sufficient to activate the kinase activity of Mec1^Rad3/ATR^. Activation depends upon physical interactions of the kinases with several activators recruited to ssDNA–RPA complexes proximal or not to primer–template regions. Dpb11^Cut5/TopBP1^ (DNA Polymerase B subunit 11/Cell Untimely Turn 5/Topoisomerase II Binding Protein 1) [161, 162], Ddc1^Rad9/RAD9^ [163], Dna2 [164] and ETAA1 (Ewing Tumor Associated Antigen 1) [165, 166] have emerged as direct activators of the apical kinases Mec1, Rad3, and ATR through physical interactions (Fig. 3b; Table 3). Dpb11^Cut5/TopBP1^ and Dna2, which participate in recruitment of DNA polymerase ε and Okazaki fragment processing, respectively, are in close proximity to regions where Mec1–Ddc2 complexes interact with stalled forks (Fig. 3b) [167]. Continued primer synthesis by Polα–Primase or discontinuous elongation of the nascent strands will also favor the recruitment of the 9–1–1 complex in proximity to the Mec1^Rad3/ATR^ complexes and will cause Mec1-dependent hyper-phosphorylation of Ddc1 [159, 168]. Phosphorylated Ddc1^Rad9/RAD9^ mediates interaction with Dpb11^Cut5/TopBP1^, facilitating Dpb11 recruitment to the DNA lesions or stalled forks and strengthening the activation of Mec1^Rad3/ATR^ (Fig. 3b) [169–171].

For full checkpoint activation, however, the signal needs to be further relayed from Mec1^Rad3/ATR^ to the downstream kinase, Rad53^Cds1/CHK1^. Outside of S phase, this process involves the 9–1–1 complex and the BRCT domain-containing factor Rad9^Rhp9/53BP1^ (Radiation sensitive 9/Rad9 homologue in *S*. *p* *ombe* 9/p53 Binding Protein 1) (Table 3) [172, 173]. Following dNTP deprivation, Rad53 activation at the stalled fork depends upon Polε and its accessory factors Dpb4 (DNA Polymerase B subunit 4), Sld2/Drc1 (Synthetically Lethal with Dpb11 2/DNA Replication Checkpoint 1) and Dpb11^Cut5/TopBP1^ [174–177]. DNA polymerase ε synergizes with Rad17–Mec3–Ddc1 and Rad9 in inducing Rad53 activation [176, 178]. Regarding this, it has been proposed that, besides Dpb11, DNA Polε is the only true activator of Mec1 following dNTP deprivation. When DNA polymerase ε functions are defective, stalled forks may become damaged, leading to the recruitment of the Ddc1–Mec3–Rad17 complex and Rad9-dependent Rad53 activation [176, 179].

**Table 3:** Direct activators and mediators of the Mec1^Rad3/ATR^–Rad53^Cds1/CHK1^ pathway upon dNTP deprivation

	*S. cerevisiae*	*S. pombe*	Human
Direct activation of Mec1^Rad3/ATR^ through physical interaction	Ddc1, Dpb11, Dna2	Cut5/Rad4	Rad9, TopBP1, ETAA1
Mediators of Rad53^Cds1/CHK1^ activation	Mrc1, Dpb4, Drc1/Sld2, DNA polymerase ε	Mrc1, Dpb4, Drc1/Sld2, DNA Polymerase ε (Cdc20)	Claspin

While Dpb11 activates Mec1 through direct physical interaction, the Polε-mediated mechanism remains elusive. Nevertheless, genetically, Dpb4, Dpb11, and Polε seem to function in the same branch of Mec1 and Rad53 activation following dNTP deprivation, suggesting that the blocked DNA polymerase ε complex on the leading strand acts as activator of the checkpoint [176, 179].

Another important step in understanding S-phase checkpoint activation was the discovery of Mrc1^Claspin^ as specific mediator for the Rad53^Cds1/CHK1^ activation upon DNA replication inhibition (Fig. 3b) [179, 180]. Following dNTP deprivation, Mrc1^Claspin^ becomes hyper-phosphorylated in a Mec1^Rad3/ATR^-dependent manner and mediates the activation of Rad53^Rad3/CHK1^ [181]. The *mrc1-AQ* allele, defective in Mec1-dependent phosphorylation, but functional with regard to replication functions in unperturbed conditions, does not support Rad53 activation following HU treatment, suggesting that Mec1-dependent targeting of Mrc1 is necessary to activate the downstream kinases of the S-phase checkpoint (Fig. 3b) [181] (Table 3). Mrc1 is associated with the replisome by means of physical interactions with the N-and C-terminal parts of Polε [182]. Mec1-dependent phosphorylation of Mrc1 abolishes the interaction with the N-terminal part of Polε [182]. Taken together these findings indicate that Mec1-dependent structural modification of the Mrc1–DNA Polymerase ε complex may lead to the formation of the true Rad53 activator at the stalled fork. In the absence of Mrc1, Rad53 activation and cellular viability, become dependent on Rad9 [181].

Future studies will perhaps continue to dissect the interplay and interactions between Mec1^ATR^, Mrc1^Claspin^ and Polε in activating Rad53^Cds1/CHK1^. As the substrates of Mec1 and Rad53 are also being unraveled during unperturbed and replication-stress conditions [183, 184], it is likely that the following years will witness increased understanding on the processes and DNA substrates that activate or are shielded by the replication checkpoint to preserve genome integrity.

### Conclusions

Studies over the past decade brought about the notion that disruption of checkpoint response pathways often underpins tumor progression, and unveiled many aspects of checkpoint function. In particular, principles underlying checkpoint activation at stalled forks, an important source of replication stress, have been put together. We have also learnt a great deal about key substrates of the S-phase checkpoint, encompassing both substrates extrinsic to replication forks and replisome components, and how their modification may affect specific cellular processes and DNA transitions at the fork. However, complicating the picture, checkpoint mutants often have pleiotropic phenotypes, making the interpretation of specific results difficult. Moreover, given the multitude of checkpoint substrates, it is likely that the checkpoint may have both activating and inhibitory roles in a specific process. Future studies will need to sort out the spatial and temporal regulations, such as those related to genomic region, chromatin state and replication timing, of checkpoint-mediated modifications, and their effect on replication proficiency and DNA dynamics during normal replication and at stalled replication forks. The interconnectedness between checkpoint activation and fork reactivation, as opposed to mere fork stabilization, has started to be investigated recently, and much remains to be learnt in this domain. Considering the recent advances in genomic, proteomic and imaging approaches, and the development of efficient and reversible conditional systems in both yeast and mammalian cells, it is certain that the following years will witness important discoveries of secret facets of the checkpoint pathway, and will unveil principles that govern the cellular response to stalled replication forks.
